# A normal motor development in congenital hydrocephalus after Cuevas Medek Exercises as early intervention: A case report

**DOI:** 10.1002/ccr3.2860

**Published:** 2020-04-27

**Authors:** Gabriela Ramires de Oliveira, Marcelo Fabris Vidal

**Affiliations:** ^1^ Physiotherapy Department at HOPE Abilitation Medical Center Dubai UAE

**Keywords:** case report, hydrocephalus, motor development, pediatrics, rehabilitation

## Abstract

Cuevas Medek Exercises (CME) harnesses optimal scientific parameters for rehabilitation programs, and this may explain its neuroplastic outcomes. A normal motor development in a high risk of delay hydrocephalus case after CME as early intervention indicates it may be considered as research subject and treatment option to prevent motor delays.

## Graphical abstract

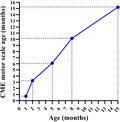

## 1 INTRODUCTION

Hydrocephalus is a disorder of cerebrospinal fluid, leading to enlargement of the ventricular system within the brain, typically associated with increased intracranial pressure. The prevalence of infant hydrocephalus is roughly one case per 1000 births. One of its most common causal mechanisms is congenital aqueduct stenosis, a focal reduction in the cerebral aqueduct at the level of the colliculus.^1^, ^2^

The effects of hydrocephalus on the developing brain involve anomalies of macro‐ and microstructure and neuronal function. Altogether, the anomalies from ventricular enlargement alter development and function of the brain both directly and indirectly. It impairs neuronal maturation, changes the cerebral vasculature, and reduces the density of capillaries in the corpus callosum, white matter, and periventricular gray matter.^2^, ^3^

Hydrocephalus conservative treatment is ventricular peritoneal shunt. Although the procedure decreases clinical signs of high intracranial pressure, as well as rapid gross restoration of the brain volume, it does not repair the axonal and neuronal damage associated with hydrocephalus.^1^ Therefore, motor deficits are frequently reported in children with hydrocephalus, which is not surprising due to the neuropathology frequently involving brain regions implicated in motor control.^1^ Hydrocephalus places individuals at risk for anomalous motor development by compromising the early development of structures central to motor function. Relative to same‐age peers, children with congenital hydrocephalus are impaired in a variety of motor skills involving posture, gait and balance, strength, and fine motor function.^1^, ^3^

In order to prevent greater developmental motor delay, a physiotherapy intervention focusing on optimizing neuroplasticity must be done, stimulating the child to achieve the developmental motor milestones. Cuevas Medek Exercises (CME) is a pediatric physiotherapy approach for children with developmental motor delay impacting the central nervous system. According to Ramon Cuevas, the creator of the method, its main principle involves provoking novel automatic motor reactions using exercises against gravity with progressive distal holdings.^4^ The method has been spread to almost all parts of the world, including well‐known rehabilitation centers in the United States, Canada, and Poland.

Our goal was to measure the effect of the CME method as early intervention in a case of congenital hydrocephalus.

## 2 CASE PRESENTATION

A 2‐months‐old Brazilian girl presented to the physiotherapy department diagnosed with congenital hydrocephalus. The hydrocephalus was detected before delivery, and she was born at 37 gestational weeks by cesarean section. No similar familiar medical history or genetic information was mentioned at the assessment.

On her first day of life, she underwent ultrasonography (US) and magnetic resonance imaging (MRI) examinations to determine the cause of the hydrocephalus. According to the US, she presented with an increase in the volume of the lateral and the third ventricles and thinning of the corpus callosum. The MRI showed a round lesion measuring 0.5 cm in the cerebral aqueduct isointense to the encephalic parenchyma without contrast enhancement, significant increase of the posterior portion of lateral ventricles, thinning of cerebral cortex, and increase of the third ventricle. At 14 days of age, she underwent implantation of a ventricular peritoneal shunt. At 2 months of age, she was assessed by a physiotherapist to initiate an intervention to prevent developmental motor delays. The assessment addressed anamnesis and a physical evaluation using the CME motor scale^4^ and the Alberta Infant Motor Scale (AIMS).^5^

The CME motor scale is composed of 41 items to assess motor development through automatic motor reactions. The response to each item is graded between 0, indicating no response, and 3, indicating complete reaction. Results provide a child's developmental motor age and can be used to create a personalized treatment plan.^4^

The AIMS is a gross motor observational tool that evaluates the control of antigravitational muscles in various postures. The AIMS is a validated scale used in clinical practice and research. It can detect developmental delays or abnormalities, being able to identify mild changes in the motor development and to measure intervention effectiveness.^5^

The assessments were conducted by an experienced physiotherapist unrelated to the intervention to avoid any influence in the outcomes. They were repeated at 3, 6, 9, and 16 months of chronological age to determine acquisition of developmental milestones and to observe treatment results. In order to interpret the outcomes properly, chronological age was corrected for prematurity.

## 3 TREATMENT

The physiotherapy intervention was composed of 1‐hour sessions once or twice per week, and daily home program exercises. The intervention was based on the CME rehabilitation method, and its main principles of provoking active movements and minimizing handling. Although developmental milestones were aimed at the specific age, more complex functions were addressed in the exercises to improve the simplest ones.^4^ Thus, in the first trimester, when head control was expected, trunk control exercises were also practiced. In the second trimester, when trunk control was expected, standing was also stimulated. When standing was expected, walking was also practiced. Our main thought was to provoke the motor development in an advanced level and prevent delays.

## 4 OUTCOMES

**Figure 1:**
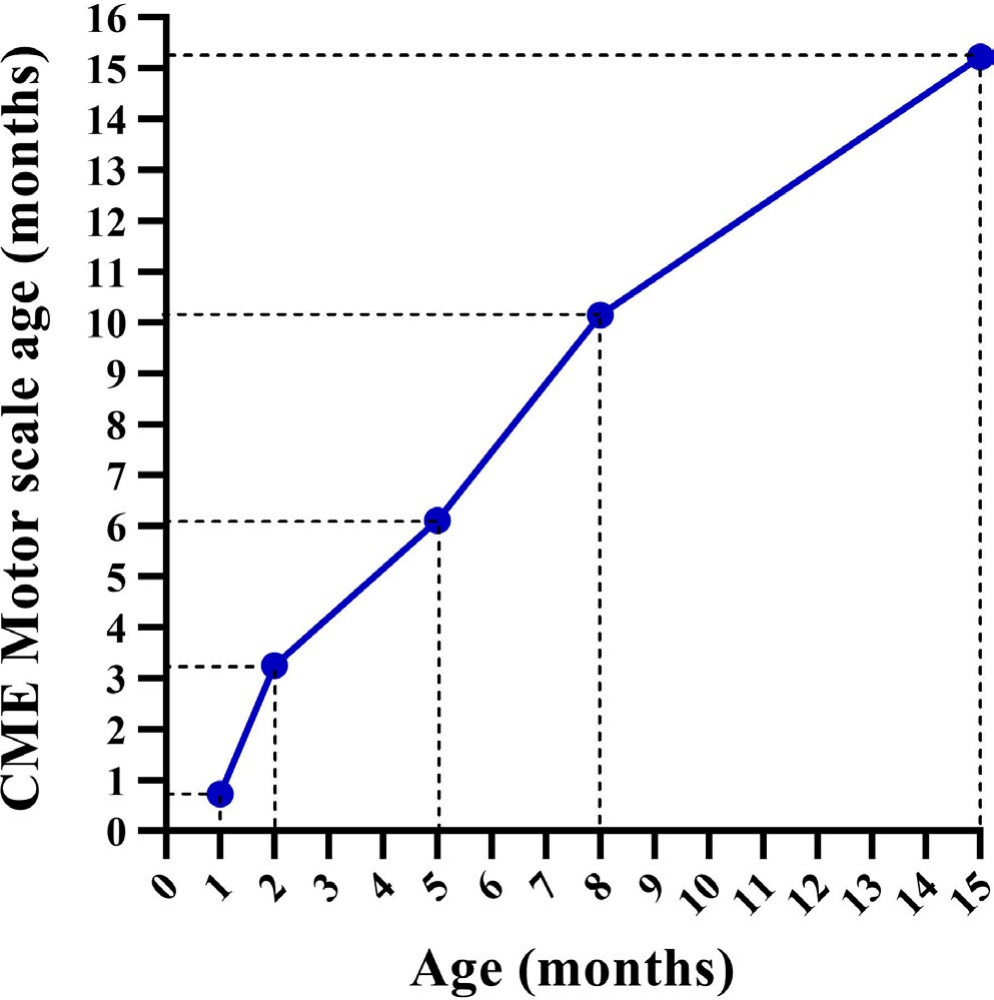
Data corresponding to developmental motor age according to CME motor scale and corrected age. CME, Cuevas Medek Exercises

According to the CME motor scale, she was considered slightly delayed at baseline. At 3, 6, 9, and 16 months of chronological age, she achieved and surpassed the motor age expected for her corrected age (Figure 1).

**Figure 2:**
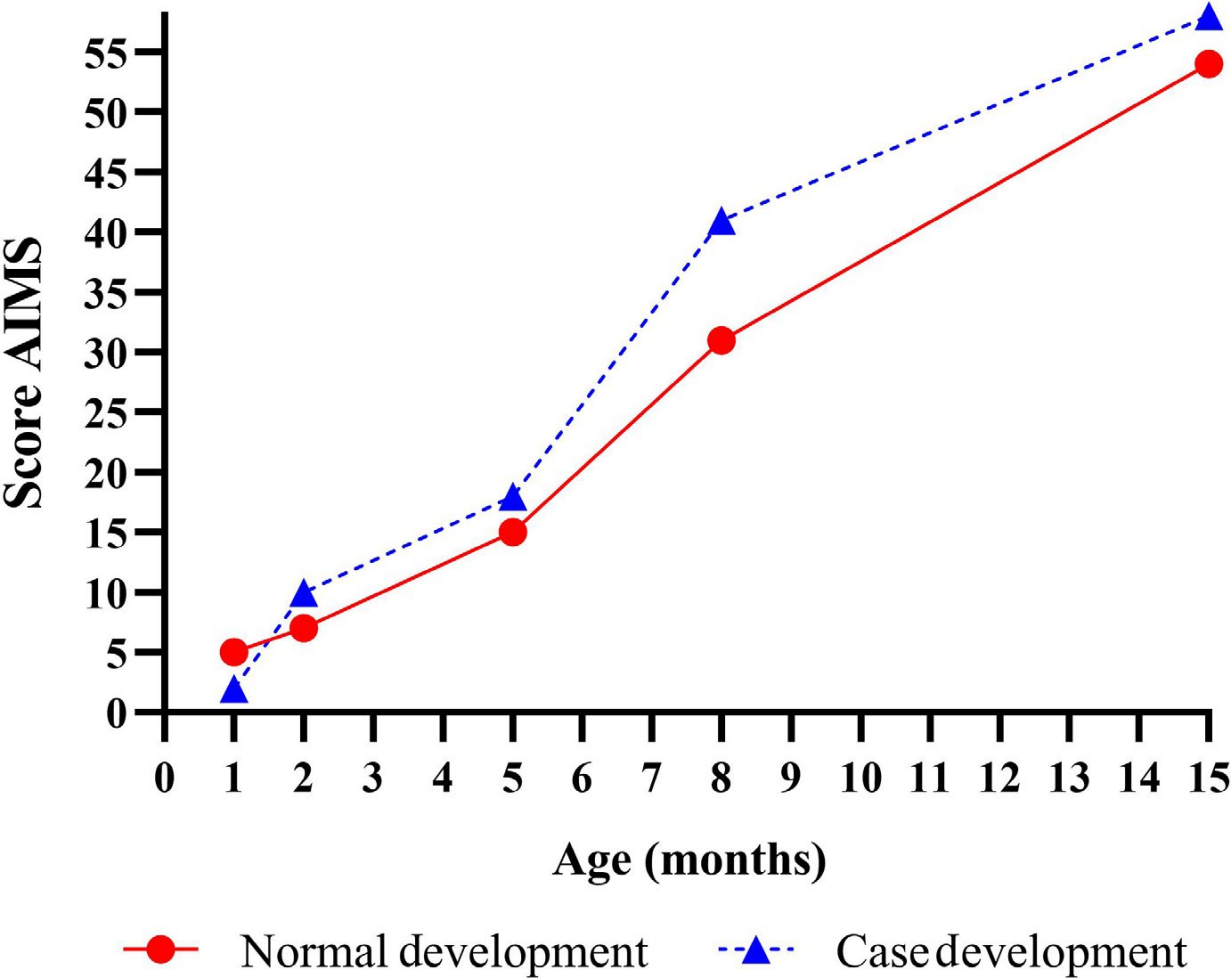
Comparison between the normal motor development curve in a Brazilian population according to AIMS^5^ and the case studied outcomes. Values below the red line are at risk of developmental motor delay. AIMS, Alberta Infant Motor Scale

According to AIMS, at baseline the child was considered to be at risk of motor delay within the normal development curve of the Brazilian population.^5^ At 3, 6, 9, and 16 months of chronological age, she was within the normal development curve for her corrected age (Figure 2).

## 5 DISCUSSION

CME was effective as an early intervention approach for preventing developmental motor delay in this hydrocephalus case, as demonstrated by these outcomes. According to CME motor scale, the early intervention based on the Cuevas Medek Exercises method was effective, considering that there were months in which the child even surpassed the level expected her corrected age. Similarly, according to the AIMS, validated and studied in the Brazilian population,^5^ CME was an effective approach for stimulating normal motor development, preventing delays in a child with hydrocephalus considered at risk of delay at the first assessment by the scale and by her pathological conditions. Also, she achieved the motor milestones at the correct age considering the windows of milestone achievement for healthy children proposed by the World Health Organization.^6^

The intervention has begun before any sign of functional delay due to her high risk of developmental motor anomalies. Earlier intervention instituted at a time of greater brain growth and plasticity is likely to be associated with a stronger beneficial effect. The field of neuroscience has repeatedly demonstrated that the plasticity of the infant brain, persistence of neurogenesis, and activity‐dependent plasticity are the basic mechanisms at work, and interventions for infants with brain injuries should aim to optimize these neuroplastic mechanisms.^7^ CME, recognized for its clinical results by therapists and parents, follows optimal scientific parameters related to neuroplasticity.

Takeuchi e Izumi^8^ showed that in order to promote neural plasticity and motor and functional recovery a rehabilitation program should include intensive, repetitive, and meaningful exercises in an enriched environment. Intensity is considered a critical factor in the rehabilitation success.^9^ Extensive movement repetition alters cortical motor representations, expanding the territory representing the repeated movement, increasing dendritic branching, synaptic growth, and response. In contrast, territories representing nonrepeated movements do not expand and may even shrink.^9^ In CME interventions, the exercises are repeated 3, 5, or 8 times, depending on the effectiveness and the quality of the expected reaction. The better the reaction, the more repetitions are done.^4^ Moreover, different and more challenging exercises should be performed to provoke the brain to react in a new way, corresponding with another important parameter for neuroplasticity, the progression. Generating and repeating novel movements, as opposed to familiar movements, are associated with the greatest neuroplastic changes. Bowdena et al^9^ found that repetition of a new pattern of movement increased the size of cortical representational areas. In contrast, repeating an already known pattern of movement did not alter the representational area.

Increasing evidence suggests meaningful exercises can assist with functional motor recovery driven by neuroplasticity.^9^ CME has thousands of different exercises to stimulate the same functional goals.^4^ While the exercises can provoke the same functional reactions, they are provided in unique ways, always creating a new challenge, out of the traditional treatment routine, making it playful and motivating. This corresponds with enriched environment (EE) interventions, which in previous studies showed that voluntary, active, playful, and challenging aspects are crucial for optimal neuroplastic outcomes.^7^

EE interventions appear to be promising to improve motor outcomes in infants.^7^, ^10^ EE is defined as an environment that facilitates enhanced cognitive, motor, and sensory stimulation. Opportunities can be provided for active motor learning, self‐generated motor activity, by adapting the physical and play environment.^7^ This approach provides greater opportunity for physical activity and motivation. The aim is to minimize handling to promote active child‐generated muscle activation and movement.^7^ Recently, a systematic review and meta‐analysis of infants at high risk of CP showed a significant effect of EE interventions on motor outcomes, suggesting that interventions including EE lead to better outcomes for infants.^10^ The main principle of CME involves consistently generating unique sensory information inside a sensorimotor challenge by minimizing handlings, moving them from proximal to distal progressively, in order to provoke a novel reaction from the brain resulting in active movements.^4^

CME as early intervention was effective in preventing developmental motor delay in a congenital hydrocephalus case. CME considers optimal scientific parameters that must be addressed in a successful rehabilitation program to promote neuroplasticity, which explains its clinical outcomes. In order to provide further support for the effectiveness of CME, additional scientific research should be done. Nevertheless, CME may be considered as a treatment option for early intervention to prevent developmental motor delay.

## CONFLICT OF INTEREST

No conflict of interest to declare.

## AUTHOR'S CONTRIBUTION

Gabriela Ramires de Oliveira: contributed to treatment applications, data collection, intellectual process, and manuscript writing. Marcelo Fabris Vidal: contributed to treatment applications, data collection, intellectual process, and manuscript writing.
